# The Flashlight Fish *Anomalops katoptron* Uses Bioluminescent Light to Detect Prey in the Dark

**DOI:** 10.1371/journal.pone.0170489

**Published:** 2017-02-08

**Authors:** Jens Hellinger, Peter Jägers, Marcel Donner, Franziska Sutt, Melanie D. Mark, Budiono Senen, Ralph Tollrian, Stefan Herlitze

**Affiliations:** 1 Department of Zoology and Neurobiology, Faculty of Biology and Biotechnology, Ruhr-University, Bochum, Germany; 2 Fisheries College Hatta-Syahrir, Banda Naira, Malukuh Tengah, Indonesia; 3 Department of Animal Ecology, Evolution and Biodiversity, Faculty of Biology and Biotechnology, Ruhr-University, Bochum, Germany; Advanced Centre for Treatment Research and Education in Cancer, INDIA

## Abstract

Bioluminescence is a fascinating phenomenon occurring in numerous animal taxa in the ocean. The reef dwelling splitfin flashlight fish (*Anomalops katoptron*) can be found in large schools during moonless nights in the shallow water of coral reefs and in the open surrounding water. *Anomalops katoptron* produce striking blink patterns with symbiotic bacteria in their sub-ocular light organs. We examined the blink frequency in *A*. *katoptron* under various laboratory conditions. During the night *A*. *katoptron* swims in schools roughly parallel to their conspecifics and display high blink frequencies of approximately 90 blinks/minute with equal on and off times. However, when planktonic prey was detected in the experimental tank, the open time increased compared to open times in the absence of prey and the frequency decreased to 20% compared to blink frequency at night in the absence of planktonic prey. During the day when the school is in a cave in the reef tank the blink frequency decreases to approximately 9 blinks/minute with increasing off-times of the light organ. Surprisingly the non-luminescent *A*. *katoptron* with non-functional light organs displayed the same blink frequencies and light organ open/closed times during the night and day as their luminescent conspecifics. In the presence of plankton non-luminescent specimens showed no change in the blink frequency and open/closed times compared to luminescent *A*. *katoptron*. Our experiments performed in a coral reef tank show that *A*. *katoptron* use bioluminescent illumination to detect planktonic prey and that the blink frequency of *A*. *katoptron* light organs follow an exogenous control by the ambient light.

## Introduction

Bioluminescence is a widespread phenomenon in nature and especially common in the oceanic environment [1–2]. Bioluminescence in the ocean exists in a wide range of genera and is most commonly found in invertebrate species. In contrast to invertebrate, vertebrates lack light emitting structures, with the exception of an abundant range of fish species that either have their own intrinsic photophore system, like hatchetfishes, dragonfishes (Stomiiformes), lanternfishes (Myctophiformes) and sharks [1–6], or host bioluminescent symbiotic bacteria in specialized light organs. Specialized light organs can be found in different fish groups like deep-sea anglerfishes [7–10], ponyfishes (Leiognathidae), e.g. *Photoplagios* [11], cardinalfishes (Apogonidae), e.g. *Siphamia tubifer* [12] and flashlight fishes (Anomalopidae), e.g. *Photoblepharon palpebratum* and *Anomalops katoptron* [13–18]. A recent study reported 27 independent evolutionary events of bioluminescence in marine ray-finned fish [19].

The family Anomalopidae comprises 6 genera including 9 species [13, 20–25]. *Anomalops katoptron*, *Photoblepharon palpebratum* and *Photoblepharon steinitzi* live in relatively shallow waters of coral reefs and can be maintained under controlled conditions in a coral-reef tank. *Anomalops katoptron* and *P*. *palpebratum* live in the Indo-Pacific region for instance in the Banda-Sea [14, 26] whereas *P*. *steinitzi* can be found in the Red Sea and the western Indian Ocean [18]. The three flashlight fishes reside in the coral reefs and show enhanced activity during moonless nights and retreat into reef caves and crevices during the daytime. Throughout dark nights *P*. *palpebratum* and *P*. *steinitzi* occur in pairs or small groups between corals and rocks [14–17] and forage zooplankton near reef caves and crevices [27]. In contrast, *A*. *katoptron* swim in schools of up to 200 specimens near the water surface. The behavioral phenomenon is described by the local Indonesian name “ikan leweri ayer,” which means “the fish which lives in the open water” [14, 17]. The splitfin flashlight fish *A*. *katoptron* is characterized by a bean shaped torch-like light organ under the eye [14]. The light organs are embedded in suborbital cavities and are connected at the anterior edge via a cartilaginous rod like attachment [14]. The suborbital light organs are densely settled with luminous symbiotic bacteria that grow in tubular structures and produce a constant bluish light [14, 16–18, 28]. The light emitted by the symbionts is enhanced by a reflector on the back of the light organ [14, 29]. *Anomalops katoptron* produce striking blink patterns during the night [14, 17]. Several functions of light organs and the blink patterns in flashlight fishes have been proposed [15, 16]: (*i*) the light organs assist in predation, i.e. to see or attract prey organisms; (*ii*) to avoid predatory fishes, i.e. evasive swimming behavior coordinated with rapid blinking (blink-and-run pattern); (*iii*) intraspecific communication, i.e. school formation, territorial behavior and courtship behavior. The blinking behavior of *Photoblepharon steinitzii* has been more extensively described in the Red Sea [15], Banda Islands [14, 17] and Comoros Islands [18]. Their light organs are nearly constantly open in the dark except for short blinks of 260 ms [15, 27]. The exact functions of the bioluminescent light for fish behavior in *A*. *katoptron* have not been analyzed.

The objective of this study was to investigate the light organ functions in the splitfin flashlight fish *A*. *katoptron* under laboratory conditions. We found that *A*. *katoptron* change its blinking pattern during the night (i.e. dark, active periods) and day (i.e. dim light, inactive periods) and during feeding. Therefore, our results strongly suggest that one function of bioluminescence in *A*. *katoptron* is the detection of planktonic prey. Additionally, we found that the blinking behavior during feeding depends on the visual system and an intact light-organ.

## Method*s*

### Field recordings on the Banda Islands

Field recordings including videos and pictures of *Anomalops katoptron* were made in the Banda Islands (Indonesia) during moonless nights by scuba diving and snorkeling using a camera in an underwater housing (Canon Powershot G15, 12 megapixel). Numbers of specimens were determined using 15 single screen shots. Video recordings were captured from different schools (n = 31; 1 recording per school) that appeared on the reef flats on Pulau Naira (n = 1 school), Banda Naira (n = 4 schools), Pulau Banda (n = 1 school) and Hatta (repeated recordings during 4 nights: n = 3, n = 5, n = 8 and n = 9 schools). In addition, videos were analyzed with respect to their schooling behavior and orientation of individual fish.

### Maintenance of fish

A school of splitfin flashlight fish (*Anomalops katoptron*) specimens were tested under different experimental conditions. All specimens were obtained from a commercial wholesaler for tropical fishes (De Jong Marinelife, Netherlands). The specimens were captured at the Cebu Island (Philippines). At least 8 months prior to the experiments, *A*. *katoptron* were kept in a coral reef tank (135 cm length x 66 cm depth x 70 cm height, 670 l volume including filter sump and macro-algae filter). For a detailed description of the reef tank, organisms, and technical equipment see S1 File. The fish were kept in a 12 h day and night cycle. The light was switched on at 1.30 am and switched of at 13.30 pm. Initially, we received 9 luminescent and 14 non-luminescent *A*. *katoptron* (see S1 File for details). 5 luminescent and 3 non-luminescent fish were used in the behavior experiments while the rest (4 luminous and 11 non-luminous fish) were kept in a separate reef tank for histological/morphological analysis. Beside the loss of luminescence non-luminous and luminous specimens displayed a similar behavior in the reef tank e.g. schooling, diurnal changes in activity patterns and feeding under dim red light. Loss of luminescence in non-luminous specimens was most likely induced by a lack of food during transport. One study reported the loss of luminescence induced by starvation [30] and we were able to restore light organ luminescence completely in specimens that arrived in the lab with nearly dark organs with high load of energy rich food after a few weeks. One third of the reef tank was protected with a non-transparent plastic panel to avoid light pollution from above. Living rocks were arranged from the bottom of the tank up to the plastic panel at the water surface to mimic a reef cave with crevices where *A*. *katoptron* seek shelter during the day. *Anomalops kataptron* were fed a mixture of frozen zooplankton organisms (5–10 mm long mysid shrimp) and lobster/fish eggs (1 mm diameter) that resemble their natural food. The frozen plankton was thawed immediately before feeding and administered in drops into the current because *A*. *katoptron* feed only moving prey organisms. During feeding the left area of the reef tank was illuminated with a red LED torch. This torch allowed the non-luminous fish to find food in the dark reef tank (see S1 File).

### Luminescence wavelength based on RGB-standard

A Red-Green-Blue (RGB) standard which shows the tonal values of red, green and blue was calculated to analyze the color (wavelength) of the bioluminescent light emitted by the light organs of *A*. *katoptron*. A monochromator (Polychrom V, TILL Photonics) was used to project a light patch on a white screen in a dark room in 10 nm steps between 380 nm and 610 nm. The projected wavelengths and *A*. *katoptron* luminescence were recorded with a HD-video camera (Sony HDR-CX 730 6.3 mm CMOS-Sensor, 24.1 megapixel, 6544 x 3680) and analyzed with ImageJ (National Institute of Health) to create a RGB-histogram standard. The three tonal RGB-values obtained from each wavelength were averaged over five repeated measurements and plotted against the wavelengths to create the RGB-standard. Light organ recordings were made in a small tank (30 x 20 x 15 cm) and analyzed as described above. The RGB values of the luminescence signal of *A*. *katoptron* was plotted in the diagram as described to determine the color/wavelength of the luminescent light. A triangle was constructed between the dissection points of light organ RGB-values and standard RGB-values. The mass centre of the triangle represents the mean wavelength of light organ emission. Red, green and blue curves represent RGB-values plotted against wavelengths. This value provides an approximation to the luminescence wavelength and not a spectral distribution.

### Anatomy of light organs

Luminous and non-luminous specimens for histological/morphological analysis were kept for at least 3 months in the laboratory. For histological analysis of light organs, 50 μm thin frozen sagittal sections were sliced and PFA (paraformaldehyde) auto-fluorescence 3D images were recorded with a Leica TCS SP5II confocal microscope. Tubule quantity in luminous and non-luminous specimens was processed with ImageJ (National Institute of Health) using grid with 300 μm side length. Six squares in each section were analyzed (see S1 File). Fishes were sacrificed with MS-222 (Sigma, Germany).

### Behavioral sampling in the reef tank

Blink frequency measurements, open and closed time analysis during the inactive period (day), active period (night) and feeding experiments (defrosted plankton/*Lysmata* larvae) were recorded using an infrared (IR) sensitive HD-video camera (Sony HDR-CX 730 6.3 mm CMOS-Sensor, 24.1 megapixel, 6544 x 3680, 30 frames/s sampling rate). Blink frequencies in non-luminescent specimens (i.e. rotation of non-luminescent light organs) were analyzed by observing the reflection of ambient light (day) and IR-light (night) in the reflector of light organs during the recordings. Recordings were conducted in the coral reef tank described above. Recordings of blinking behavior (frequency and open/closed times) were made during the active period (night) and inactive period (day). Active period (night) was characterized by increased swimming activity across the whole tank volume of both luminous and non-luminous fish, schooling behavior and increased blink frequency. The school exhibits a normal activity pattern at this time (preliminary observation and field observations). Inactive period (day) was characterized by reduced movements in the artificial cave and reduced blink frequency during the day in both luminous and non-luminous specimens. Blinking behavior during their active period (night), feeding with frozen zooplankton and feeding with cleaner shrimp (*Lysmata amboiensis*) zoea larvae were recorded through the front of the reef tank. Blinking behavior during the inactive period (day) was recorded through a window, covered with a removable plastic door, on one side of the reef tank, which allowed sight into the cavity where *A*. *katoptron* seek shelter in the daytime. The reef tank was illuminated by infrared (IR) LED lamps with 850 nm wavelength (Abus, Germany) during night recordings. All specimens of *A*. *katoptron* were tested for IR-insensitivity during preliminary observations and showed no reaction to the on-/offset of IR-illumination. Furthermore non-luminous specimens were unable to find food items during the night under IR-illumination. Specimens were identified by body length, fin margins, light organ size and non-luminous patches on the light organ. Fish behavior in the inactive period (day), active period (night) and during feeding defrosted plankton were recorded in sequential sessions in fixed sample time intervals (5 min per session, 1 session per day on 5 subsequent days). When specimen moved out of focus the recording was stopped and repeated. Feeding with natural zooplankton (*L*. *amboiensis* larvae) was recorded 2 times for 5 minutes to analyze blink frequency, open/closed time and feeding performance during decreasing larvae density. Data sampling was started directly after hatching began. Video recordings were analyzed frame by frame for each specimen of tested *A*. *katoptron* with the video analysis software VidAna.

### Experiments during the inactive period (day)

Experiments under dim light conditions were performed during the day to investigate the behavior in the inactive period (day) and when weak light was present in the artificial reef cave. The experiments were started 6 hours the after the tank illumination was switched on (see above). Inactive periods (night) were described above. Experiments were performed under 3 dim light conditions (dl) with a decrease in light intensity from dl i3 to dl i1 (dl i3: 0.63 μW/cm^2^, dl i2: 0.134 μW/cm^2^, dl i1: 0.026 μW/cm^2^). Light intensities were recorded with a optical power meter (Pm100D & S120C photodiode sensor Thorlabs, US) in the artificial cave. The mean light intensity was recorded at 3 points in the artificial cave where *A*. *katoptron* reside during the day. Experiments with light intensity i3 were recorded in the camera’s daylight mode. Experiments with light intensities i1 and i2 were recorded under IR-illumination because the intensities were not bright enough for the camera sensor in normal daylight mode. The test set-up was covered with a light-tight cloth to avoid the influence of room light. Recordings were started 3 h after setup installation and 3 h before before the tank illumination were switched off (see above) via a remote control.

### Experiments during the active period (night)

Experiments during the night were conducted 2 h after the tank illumination was switched off (see above). Active period (night) was defined as mentioned above. The camera was mounted on a tripod in front of the tank. The tank was illuminated by IR-light during darkness experiments as described above. Recordings were performed using a remote control.

### Feeding experiments with frozen plankton organisms during active periods (night)

Feeding experiments were implemented during active periods in the night. Active periods (night) were defined above. All specimens were fed under illumination of a red LED (see above), illuminated in the left corner of the reef tank. The red light allowed non-luminous specimens to find moving food particles in the water current. A defrosted plankton mixture (mysid shrimp 5–10 mm length and lobster/fish eggs 1 mm diameter) was applied dropwise to the water column in approximately 5 s intervals. This resulted in a total volume of approximately 150 ml of defrosted zooplankton. Preliminary feeding tests were performed to avoid satiation effects. The school *of A*. *katoptron* was fed with 200 ml of defrosted zooplankton continuously in consecutive sessions without any signs of repletion.

### Feeding experiments with natural plankton (cleaner shrimp larvae) during active periods (night)

Feeding experiments with natural zooplankton were performed to test the function of light organs under hunting conditions at night when *A*. *katoptron* display increased activity. Four adult cleaner shrimps (*Lysmata amboiensis*) were kept together with *A*. *katoptron*. Adult cleaner shrimps release zoea-larvae (2.7–2.8 mm in length [31]), during the night. Individual spawning of *L*. *amboiensis* was predicted by monitoring the abdomen. If eggs were visible, recording experiments were started on consecutive days until the cleaner shrimp larvae started hatching. The zoea-larvae displayed saccadic movements in the water current. Recordings were performed under IR-illumination as mentioned above. Recordings were started directly after larval hatching. Blink frequency, open & closed time of light organs and feeding performance were compared for specimens with luminous and non-luminous light organs.

### Statistical analysis

SigmaPlot 12.0 was used to analyze data from behavior experiments. Subject differences and group comparison in blink frequency, open & closed times of light organs and prey capture (*L*. *amboiensis* larvae) were tested using a one way repeated measures ANOVA and Holm-Sidak post hoc analysis. Differences in tubule quantity were analyzed via Mann-Whitney rank-sum test. Results are reported as mean ± SEM (standard error of mean). Significance values were represented as follows: *P ≤ 0.05; **P ≤ 0.01; ***P ≤ 0.001. All experiments were approved by the Institutional Animal Research Facility, University of Bochum, Germany and LANUV, NRW, Recklinghausen, Germany. All methods were carried out in accordance with the approved guidelines.

## Results

### Field recordings of bioluminescent *A*. *katoptron* on the Banda Islands

**Fig 1:**
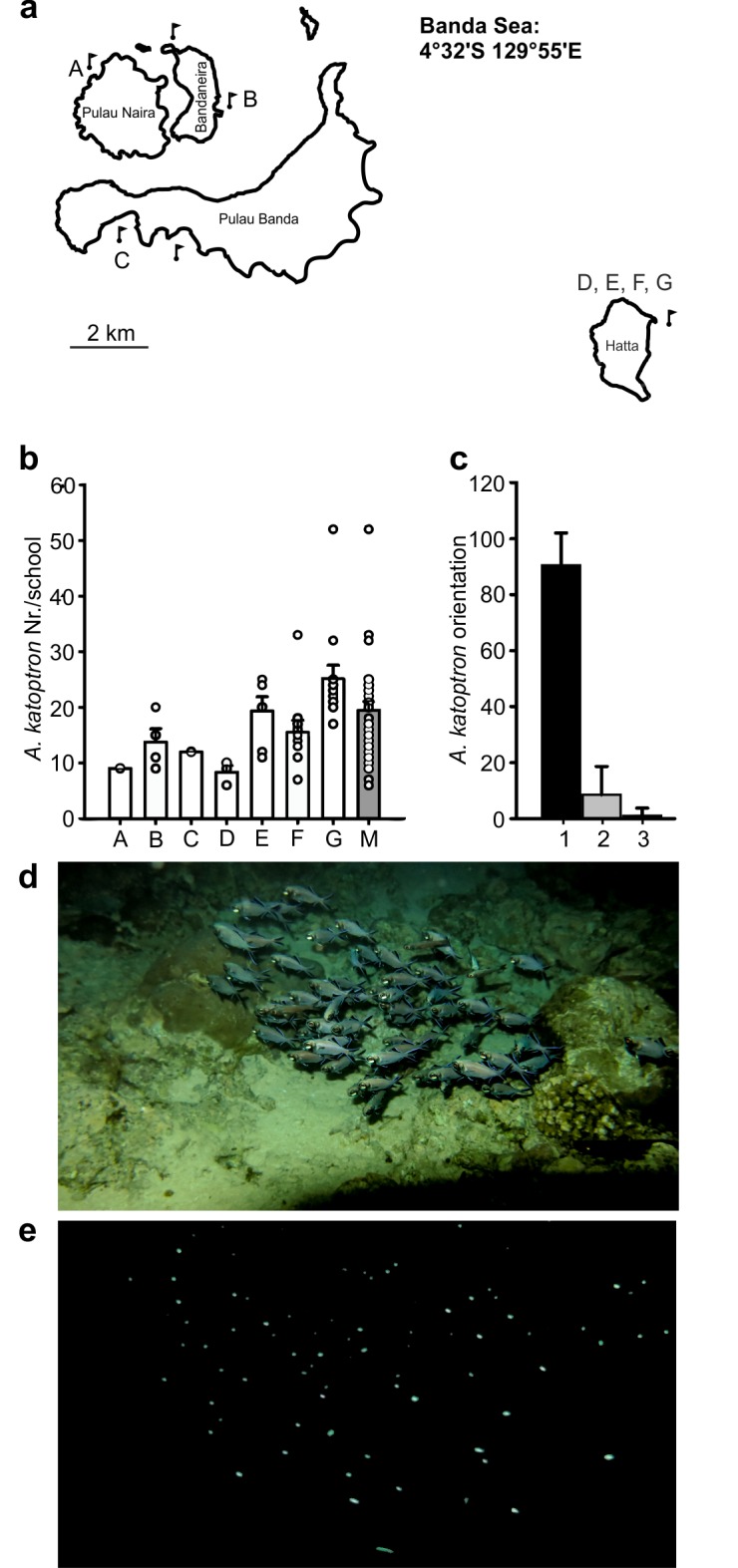
Field recordings on the Banda Islands nights. (a) Observation sites marked by black burgees. Samples are indicated by A-G. Map adapted from OpenStreetMap-contributor (Open Database Licence (ODbL) 1.0). (b) Number of individuals in schools at different study sites A: Pulau Naira, B: Banda Naira, C: Pulau Banda, D-G: Hatta. Circles indicate individual schools. A total of 31 schools (1 recording per school) were recorded at the study sites. (M) represents the mean value of fish per school ± SEM. (c) Orientation of schooling *A*. *katoptron* on the coral reef flat during the night. Pictures were recorded with an internal camera flash. Black bar (1) indicates specimens orientated in one direction (see example in c). Grey bar (2) indicates the opposite direction. White bar (3) indicates specimens in perpendicular orientation. (d) Schooling behavior in *A*. *katoptron* on a reef flat during a moonless night. Flash photograph of an *A*. *katoptron* school showing the majority of fish moving in the same direction, and a minority positioned in the opposite or perpendicular direction. (e) Bioluminescence of *A*. *katoptron* light organs in complete darkness Error bars indicate ± SEM.

In 1909 Steche [14] and in 1971 Haneda and Tsuji [17] described that *A*. *katoptron* can be found a short distance from the shores of the Banda Islands in Indonesia. In order to determine the school size and schooling behaviour of *A*. *katoptron*, we recorded schools of *A*. *katoptron* during moonless nights at four different sites near the Banda Islands (Fig 1A). School size varied from 8 to 50 specimens (Fig 1B & S1 Movie). The schools came very close (less than 1 m) to the water surface both on the reef flat and farther away from the reef crest. The majority of *A*. *katoptron* within the schools were orientated in the same direction (Fig 1C and 1D) producing luminescent signals (Fig 1E).

### Light emission and anatomy of light organs

**Fig 2:**
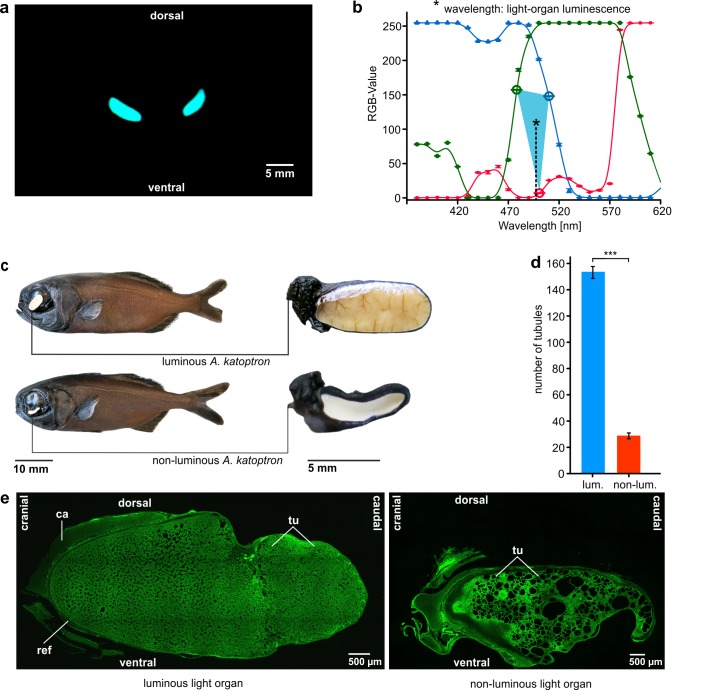
Bioluminescence, anatomical position and structure of the light organs in the splitfin flashlight fish (*Anomalops katoptron)*. (a) Bioluminescence of *A*. *katoptron* during the night. Front view of both subocular light organs. The photograph was taken in a reef tank during the night. (b) Approximated bioluminescence wavelength (498 nm) of the emitted light. (c) Habitus, subocular position and structure of the light organ of the splitfin flashlight fish (*Anomalops katoptron*) shown for one luminescent and one non-luminescent specimen with degenerated light organs. The oval light organ appears as a white patch because of the guanine crystal reflector on the backside of the light organ and photography using a camera flash. The degenerated non-luminescent light organ illustrates a loss of blood vessels on the surface and a change in shape. (d) Number of tubules in luminous (n = 4) and non-luminous (n = 11) specimens of *A*. *katoptron*. Error bars indicate ± SEM (e) Sagittal 3D-photomicrographs of one luminescent and one non-luminescent light organ. The images show the tubules (tu) where *A*. *kataptron* host the bioluminescent bacterial symbionts, the reflector (ref), and the cartilaginous light organ attachment (ca) located at the frontal apex.

The light organs from luminescent specimens display a bright luminescent light (Fig 2A). Analysis of light organ RGB-values, compared to narrow-band light source standards, revealed a bioluminescence wavelength of around 500 nm for *A*. *katoptron* light organ luminescence (Fig 2B). Macroscopic light organ anatomy in luminescent *A*. *katoptron* (n = 4) displayed normal bean shaped light organs with a high amount of blood vessels at the surface. In contrast, non-luminous specimens revealed degenerated light organs and a loss of blood vessels while the body shape and length of luminous (9.25 cm ± 0,3 SEM) and non-luminous (9.29 cm ± 0,3 SEM) specimens showed no obvious differences (Fig 2C). The microscopic anatomy of luminescent light organs showed a regular pattern of densely packed tubules where *A*. *katoptron* host symbiotic luminescent bacteria, a cartilaginous attachment on the frontal apex and a reflector as described by Steche (1909) [14] and Bassot (1968) [28]. Non-luminous light organs were degenerated and characterized by a decrease in overall shape and tubule density (Fig 2D) and an increase in large open spaces. (Fig 2E).

### Blinking behavior in the reef tank during day and night conditions

**Fig 3:**
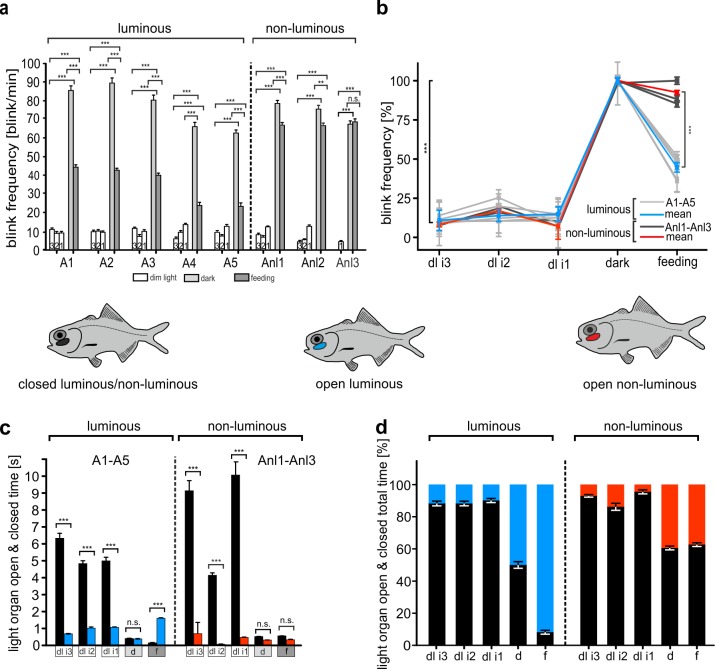
Blinking behaviour in *A*. *katoptron* under graded dim light conditions, in darkness and while feeding defrosted zooplankton in darkness. (a) Blink frequency (blinks/min) in *A*. *katoptron* with luminous light organs (A1-A5) and non-luminous light organs (Anl1-Anl3). Luminous and non-luminous fish rotated the light organs with a comparable frequency under 3 decreasing dim light conditions. White bars (1–3) indicate blink frequencies under dim light (i3-i1) conditions. Light grey bars indicate blink frequencies at night. Dark grey bars indicate blink frequencies while feeding frozen zooplankton. (b) Summarized percentiles of blink frequencies in *A*. *katoptron* with luminous (solid grey lines: A1-A5, n = 5; blue line: average A1-A5) and non-luminous light organs (dark grey lines: Anl1-Anl3, n = 3; red line: average Anl1-Anl3) under the conditions dim light i1-i3, dark and feeding with frozen zooplankton in the dark. (c) Mean time intervals based on single open & closed times of light organs during dim light (dl i1-dl i3; white squares), darkness (d, light grey squares), and feeding in the dark (f, dark grey squares) conditions. (d) Total relative distribution of open & closed light organs during dim light (dl i1-dl i3), darkness and feeding in the dark experiments. Icons illustrate color coding in c-d. Black indicates closed light organs for both luminous and non-luminous light organs. *Blue* indicates luminous open light organs. *Red* indicates non-luminous open light organs. Error bars indicate ± SEM.

We first analyzed the blinking behavior of *A*. *katoptron* during the daily inactive period (dim light, dl; S2–S4 Movies) in comparison to the active period (night) in the reef tank. We tested the blinking behavior under three different levels of weak illumination in the artificial reef cave and observed similar low mean blink frequencies (dl i3: 11.5 ± 0.54 blinks/min; dl i2: 8.5 ± 0.4 blinks/min; dl i1: 10.8 ± 0.41 blinks/min (Fig 3A)). The average blink frequency in *A*. *katoptron* increased from 13.8 ± 1.2% during dim light conditions to 89 ± 2.7 blinks/min during the night (ANOVA, F = 82.76, P < 0.001; Holm-Sidak, P < 0.001). We defined the 89 blinks/min as 100% (Fig 3A and 3B). Specimens without luminescent light organs displayed blink frequencies under both dim light and dark conditions comparable to those of luminescent fish (Fig 3A and 3B).

### Open and closed times of light organs during day and night conditions

We next investigated how long the light organs were open and closed during day and night. We found that in the inactive day period (dl i1-dl i3) light organs were closed for around 6 s and open for around 1 s for both luminous and non-luminous fish (Table 1). In contrast, under dark conditions the single closing and open times decreased (ANOVA, F = 58.25, P < 0.001; Holm-Sidak, P < 0.001) for both luminous and non-luminous fish (Fig 3C, Table 1). We next calculated the relative percentage of how long the light organs were open and closed during the 5 min recording interval. During the inactive period (day) light organs in luminous fish were closed for around 90% and open for around 10% of the time (see S1A Fig for absolute values). In contrast during the active period (night) light organs were open and closed approximately for the same amount of time (Fig 3D). Thus, we observed no significant differences in open and close times for the light organs in luminous and non-luminous fish during the inactive daily period and the active nightly period (Fig 3D & see S1 Fig for absolute values). All specimens with luminous or non-luminous light organs displayed enhanced swimming activity in darkness (S5 Movie) comparable to the behavior observed in the field (S1 Movie).

**Table 1:** Comparison of single light organ open/closed times for luminous and non-luminous *A*. *katoptron*.

		day*		night		feeding	
		luminous	non-luminous	luminous	non-luminous	luminous	non-luminous
closed [s]	**min.**	0.033	0.045	0.04	0.05	0.04	0.045
	**mean**	5.549	9.649	0.41	0.523	0.149	0.558
	**max.**	198.37	92.36	8.4	6	3.12	3.28
	**± SEM**	0.179	0.493	0.005	0.078	0.033	0.005
open [s]	**min.**	0.018	0.448	0.08	0.05	0.08	0.08
	**mean**	0.917	0.586	0.383	0.318	1.602	0.332
	**max.**	17.88	7.6	5.68	2.8	36,32	2.24
	**± SEM**	0.018	0.023	0.003	0.003	0.009	0.025

min., minimum time; max., maximum time; mean, mean value; SEM, standard error of the mean

*synopsis of 3 light levels (dl i1-dl i3)

### Blinking behavior while feeding defrosted plankton

The defrosted plankton was distributed throughout the tank by the water current and eaten by both luminous and non-luminous *A*. *katoptron* (see S6 Movie). During feeding luminescent *A*. *katoptron* displayed a 44.7 ± 3.04% decrease (ANOVA, F = 82.76, P < 0.001; Holm-Sidak, P < 0.001) in blink frequency compared to blink frequencies under dark conditions (defined as 100%) without plankton as a control (Fig 3B). In contrast, the non-luminescent fish demonstrated no shift in blink frequency during feeding. Thus, the blink frequency in non-luminescent fish was significantly higher (ANOVA, F = 30.895, P < 0.001) than in luminescent specimens during feeding (Fig 3B). The relative single opening times of the light organs in luminescent specimens were augmented under dark and more dramatically under feeding conditions compared to dim light conditions (Fig 3D). The mean open time (1.6 ± 0.03 s) was significantly (ANOVA, F = 26.619, P < 0.001) increased (Fig 3C) compared to the mean closed time (0.15 ± 0.03 s). In contrast non-luminescent *A*. *katoptron* showed no change in mean open or close times during feeding (Fig 3C). Light organs were open for 91.7 ± 0.96% during the recording session in luminous specimens compared to only 37.2 ± 1.43% in non-luminescent fish (Fig 3D). Thus, luminous fish but not non-luminous fish decrease their blinking frequencies and increase their total light emission during feeding.

### Blinking behavior while hunting living prey

**Fig 4:**
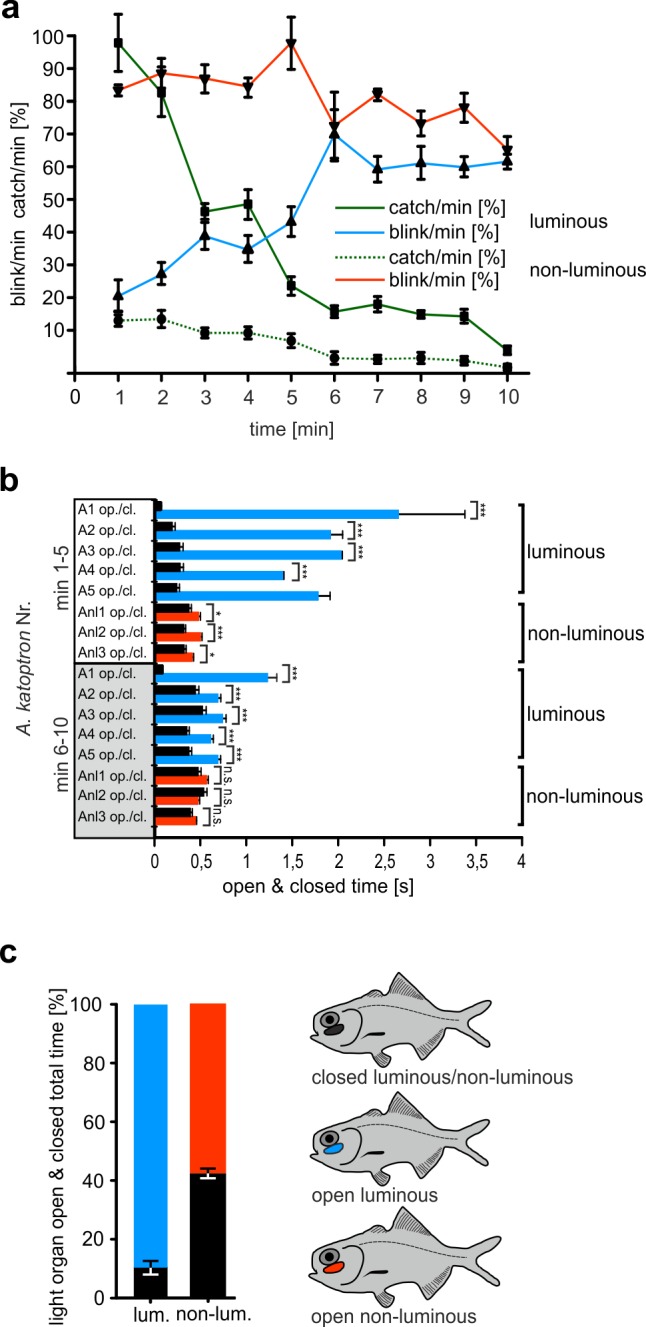
Blink frequency and catch efficiency in *A*. *katoptron* feeding on currently hatching cleaner shrimp larvea (*L*. *amboiensis*). (a) Blink frequency percentiles for specimens with luminous (blue line A1-A5, n = 5) and non-luminous (red line Anl1-Anl3, n = 3) light organs during 2 subsequent 5 minute recording sessions (minute 1–5 & minute 6–10) to illustrate the changes during decreasing larvae density. (b) Average open & closed time of light organs for individual specimens with luminous light organs (A1-A5, n = 5) and non-luminous light organs (Anl1-Anl3, n = 3). Pooled data from two subsequent observation periods, (min 1–5: directly after hatching; min 6–10: observation period directly after min 1–5) each of five minutes. (c) Percentile distribution of open & closed times of light organs in *A*. *katoptron*, summary of the 10 min feeding period. Icons illustrate color coding for bar graphs 4b and 4c. Icons illustrate color coding in c. Black indicates closed light organs for both luminous and non-luminous light organs. *Blue* indicates luminous open light organs. *Red* indicates non-luminous open light organs. Error bars indicate ± SEM.

We next investigated how the blink frequencies and open and closed times of light organs in luminescent and non-luminescent *A*. *katoptron* were influenced by the presence of living natural prey organisms (S7 Movie). Blink frequencies during their active period (night) without food were defined as 100% (Fig 4A). Luminous *A*. *katoptrons* immediately decreased (ANOVA, F = 58.074, P < 0.001) their blink frequency to 18.6 ± 4.7% after hatching started (Fig 4A) and increased the open times of light organs with no change in closed times (Fig 4B). During the 5 min recording time the light organs were open for 89.5 ± 2.3% and closed for 10.5 ± 2.3% (Fig 4C). The decrease in blink frequencies and increase in the open time of the light organ was correlated with the maximum number of caught shrimp larvae (70.6 ± 6.5 catch/min, defined as 100%) (Fig 4A). As the number of shrimp larvae and catches decreased over time, we observed a concomitant increase of blink frequency over time. No change in the blink frequency was observed in non-luminescent *A*. *katoptron* after shrimp larvae hatching and only approximately 10% of the shrimp were captured when compared to luminescent *A*. *katoptron* (Fig 4A). The experiments suggest that an increase in luminous light emission of *A*.*katoptron* by decreasing blink frequencies and increasing the open time of the light organs is used to detect and catch prey.

## Discussion

Very little information is available on the role of the light organ of *Anomalops katoptrons* for the behavior. In 1975 Morin suggested several functions of light organs in *Photoblepharon steinitzii* for example in assisting predation, avoiding predation and intraspecific communication based on observations in the field and laboratory [15]. The splitfin flashlight fish *A*. *katoptron* live together with *P*. *palpebratus* in Indonesia e.g. the Banda Islands [14, 26]. Fast blinking activity in *A*. *katoptron* was reported by different authors primarily based on observations in the field [14, 26] and from specimen individually housed in glass jars [17] or from unpublished data by Morin which described high blink frequencies up to 116 blinks/min [27]. In this article we present the first quantitative study on the light organ blinking activity in the splitfin flashlight fish *A*. *katoptron* during the day/night cycle and during hunting under controlled laboratory conditions. The light organs in *A*. *katoptron* emit blue/cyan light with a wavelength of approximately 500 nm. Light emission in the blue range around 470 nm is common among many luminescent organisms [2]. Furthermore, we demonstrate that a loss of luminescence in *Anomalops* is followed by a macroscopic change in light organ anatomy and a reduction of the microscopic regular tubular structure described by Steche and Bassot [14, 28]. Interestingly, the anatomical change had no impact on the complex light organ rotation mechanism in *A*. *katoptron* [32]. The loss of luminescence combined with an anatomical change underlines the mutualistic relationship between the vertebrate host *A*. *katoptron* and the bacterial symbiont '*Candidatus* Photodesmus katoptron' [33].

### Blinking behavior during day and night conditions

We found in our study that the splitfin flashlight fish *A*. *katoptron* alter their blink frequencies and light organ opening times in a context dependent manner. Our data show that a school of *A*. *katoptron* reduces the blink frequency and decreases the opening times of light organs during the day under dim light conditions in an artificial cave. Haneda described that specimens of *A*. *katoptron* closed their light organs when individually housed in a glass jar and lighted room [17]. Thus, it appears that the backward rotation of the light organ is triggered by light. Unfortunately, the separation of schooling fish in a bare glass jar and exposure to room light provides little information about the natural blinking behavior during the day. *Anomalops katoptron* seek shelter in regions below coral reefs and can be found deeper than 60 m during the day [27]. Therefore we investigated the behavior of *A*. *katoptron* under 3 decreasing dim light levels (see S2–S4 Movies) to mimic residual light conditions in the reef tank. Under dim light (dl i3-dl i1) conditions *A*. *katoptron* reduce their swimming activity, blinking frequency and seek shelter in the artificial reef cave. The light organs are closed for long periods of time interrupted by short blinks. One reason that the light organs are closed during the day potentially relied on the fact, that the backside of light organs in *A*. *katoptron* is dark colored like the body surface. Furthermore, the inside of the light organ is enclosed by a guanine crystal reflector (Fig 2C & 2E) as described by Steche and Bassot [14, 28]. During the night the reflector enhances the efficiency of bacterial luminescence of light organs [29]. Under dim light conditions, however, the mirror reflects the ambient light and appears as a bright white spot against their dark body surface. This immense visual contrast between their dark bodies and bright, exposed light organs may increase *A*. *katoptron* vulnerability for predators [34]. Therefore, we propose that reduced blink patterns under dim light conditions (during the day) potentially reduce predation risk, by reducing the visual contrast between the dark body surface and bright light organ. Although the blinking activity of the light organ under dim light conditions was measured in an artificial system that mimics their natural surroundings, it is likely that *A*. *katoptron* display a similar behavior in the field because residual light from the sun also travels deep in the clear water of coral reefs. For example phytoplankton in mesophotic coral ecosytems (1–150 m) can be found up to 91 m [35] and the photosynthesizing coral *Leptoseris fragilis* up to 145 m water depth [36]. Therefore, further field studies have to show if schools of *A*. *katoptron* display the same behavior under natural conditions in their daytime shelters.

During the night *A*. *katoptron* display high blink frequencies with increased light on times (Fig 3A and S3 Movie). The production of light signals in a dark environment increase the predation risk for small planktivorous species, which lack defensive structures like sharp spines. *Anomalops katoptron* occurs in large schools up to 200 individuals (Fig 1) [14, 16–17]. Therefore high blinking frequencies of individual fish in a school, which frequently changes its swimming direction, can distract potential predators and potentially increase the benefits of schooling behavior, e.g. safety and foraging [34, 37–38]. While foraging is an offensive function of bioluminescence, safety is defensive [2]. Defensive functions were described for several fish species, e.g. ventral counter illumination in leiognathid fish [39] and bioluminescent sharks [40]. Morin described a “blink and run” behavior in *Photoblepharon* where they display a rapid blink pattern associated with a darting swimming profile [15] for potentially distracting predators.

### Open and closed times of light organs during day and night condtions

The light organs in *A*. *katoptron* are open and closed for approximately equal times during the night. In contrast the light organ in the related flashlight fish *Photoblepharon palpebratus* is open most of the time during the night interrupted by short off times [15]. The differences between light organ open times in *Photoblepharon steinitzii* and *Anomalops katoptron* are probably related to a different nocturnal behavior. *Anomalops* swim in schools on the reef and in the open water far away from their daytime shelter and display a high frequency blink behavior, whereas *Photoblepharon* live in the vicinity of shelter and display mostly open light organs [15]. This possibly allows an easier detection of prey at the cost of higher risk to be attacked by predators. *Photoblepharon* can quickly seek shelter in reef caves and crevices while predators appear. In contrast schools of *A*. *katoptron* probably distract predators with their high blink frequencies in unprotected areas during the night. The high blink frequency with roughly equal open and closed times could be a tradeoff between seeing planktonic prey, intraspecific communication, substrate illumination and the risk of detection by nocturnal predators. Thus high blink frequency and equal on and off times could maximize the benefits and minimize the risk of light production. The fact that non-luminous specimens displayed the same high blink frequency pattern during the night suggests that the blink behavior is influenced by the ambient lighting conditions (darkness) during the active period (night) of *A*. *katoptron*.

### Blinking behavior during hunting prey

Prey capture and lure were described as a function of bioluminescence for several bioluminescent fish species. Morin described that the related species *P*. *steinitzi* illuminated *Artemia* with the luminescent light organ and suggested that the light attracts planktonic crustaceans during the night [15]. McCosker suggested that the change in blinking behavior in *P*. *palpebratus* informs conspecifics about the presence of prey [16]. Prey capture was also suggested for non-beryciform species. The leioghnatid fish *Gazza minuta* display a discrete projected luminescence and McFall-Ngai proposed that *G*. *minuta* attract and locate the fish on which *G*. *minuta* nocturnally feeds [39]. The famous bioluminescent deep-sea anglerfishes display an escal photophore and are generally thought to attract prey with this lure [27]. The apogonid fish *Siphamia tubifer* was also suggested to use their ventral light organ to search and attract prey [39, 41]. Interestingly *Siphamia tubifer* show a different activity and feeding strategy compared to *Anomalops* and *Photoblepharon*. *Siphamia* seems to use the light organ to find plankton only at twilight and not in darkness during the night [41].

Contrary to the blink pattern during the night, *A*. *katoptron* reduced their blink frequency during feeding with increased light-on times to provide more light when feeding on natural living prey and defrosted zooplankton. Our feeding experiments show that the splitfin flashlight fish *A*. *katoptron* use their light organs to search and detect prey in the dark. Furthermore this predatory behavior seems to be controlled by the visual and light organ systems to optimally detect prey with their own bioluminescent light source. More importantly, only luminous *A*. *katoptron* displayed a high feeding performance and change in blinking behavior but not non-luminous specimens. This implies that the detection of prey organisms with their own visual system and light organs trigger the changes in blink behavior and the increase in opening times. Non-luminous *A*. *katoptron* showed no difference in size compared to luminescence fish because they were able to find food under red light conditions during the normal daily feeding. This observation leads to the conclusion that non-luminous *A*. *katoptron* are capable of hunting without bioluminescence under artificial illumination. Most fish have efficient visual abilities [42] and our data show that *A*. *katoptron* is able to detect plankton in a dark environment with the luminescent light. Once prey is detected *A*. *katoptron* change their high frequency blinking behavior to a constant glow, which allows a high feeding performance, most likely because there is more light to see the prey. The disadvantage of a constant glow during the night is that *A*. *katoptron* is more vulnerable to predators. The decrease of blinking frequency and increase in open time of the light organ during feeding is only detected in luminous specimens but not in non-luminous specimens. The change from fast blinking to constant glowing is most likely triggered by the visual system and not by intraspecific communication otherwise non-luminescent specimens would also change the blink frequency in the presence of prey. Furthermore our results suggest that light organ of *A*. *katoptron* is probably used to illuminate rather than attract prey organisms. The fast blinking and swimming behavior would make it difficult for plankton to reach a school of flashlight fish in contrast to bioluminescent fishes that live in a more circumscribed area [14, 15] and produce a nearly constant light and short blinks such as *Photoblepharon* [15]. Our results may also suggest a role for interspecific competition with non-luminescent nocturnal planktivorous fish e.g. Apogonidae and Myripristinae, but further studies are necessary to show for example differences in feeding performance and growth rates between luminescent flashlight fishes and non-luminescent nocturnal fishes. Interspecific competition was described for a range of fish species [43] such as gobies *(Gobiodon*) that inhabit coral reefs [44].

In summary our results lead to 3 conclusions: (1) *A*. *katoptron* change their blink behavior in darkness to use their light organs as a light source to illuminate and find prey organisms. (2) The behavioral shift in blink frequency and open and closing times of light organs in the dark is triggered by the visualization of prey through their own light. Furthermore our data suggest that the behavioral shift in blink frequency and open and closed times during feeding is not triggered by intraspecific communication. The visual system appears to be the predominant sense during feeding. This is also indicated by the fact that non-luminous fish are capable to forage under red light conditions, which is impaired in darkness (Fig 4A). Other sensory systems e.g. the lateral line and the olfactory system most likely play only a minor part as indicated by experiments with non-luminous *A*. *katoptron*. (3) The blink pattern in *A*. *katoptron* during their night and day cycle is influenced by the ambient light and displays an exogenous control by the ambient light.

## Supporting Information

S1 File Supporting Information. (DOCX)

S1 Fig Blink behaviour in *A*. *katoptron* during the day (3 dim light conditions), night (darkness) and feeding in darkness (frozen plankton). (TIF)

S1 Movie Schooling behaviour Banda Islands. (AVI)

S2 Movie Blink frequency dim light dl i3. (AVI)

S3 Movie Blink frequency dim light dl i2. (AVI)

S4 Movie Blink frequency dim light dl i1. (AVI)

S5 Movie Blink frequency darkness. (AVI)

S6 Movie Blink frequency frozen plankton. (AVI)

S7 Movie Blink frequency feeding cleaner shrimp larvae. (AVI)
