# Supplementary material for: The Flashlight Fish Anomalops katoptron Uses Bioluminescent Light to Detect Prey in the Dark
Source: PLoS One. 2017 Feb 8;12(2):e0170489. doi: 10.1371/journal.pone.0170489 (PMC5298212; doi:10.1371/journal.pone.0170489)
Supplement: S1 File — (DOCX) [file pone.0170489.s001.docx]

**Supporting Information**

**Title: The flashlight fish *Anomalops katoptron* uses bioluminescent light to detect prey in the dark**

Jens Hellinger^1^, Peter Jägers^1^, Marcel Donner^1^, Franziska Sutt^1^, Melanie D. Mark^1^, Budiono Senen^2^_,_ Ralph Tollrian^3^ and Stefan Herlitze^1*^

^1^Department of Zoology and Neurobiology, ND7/31, Ruhr-University Bochum, Universitätsstr. 150, D-44780 Bochum, Germany

^2^Fisheries College Hatta-Syahrir, Jl. Said Tjong Baadilla No 1, 97593 Banda Naira, Malukuh Tengah, Indonesia

^3^Department of Animal Ecology, Evolution and Biodiversity, NDEF 05/754, Ruhr-University Bochum, Universitätsstr. 150, D-44780 Bochum, Germany

*Corresponding author

E-mail: [sxh106@gmail.com](mailto:sxh106@gmail.com); stefan.herlitze@rub.de

**Maintenance of *A. katoptron***

A shoal of 23 adult (9-12 cm body-length) splitfin flashlight fish (*Anomalops katoptron*) Cebu Island (Philippines) was obtained from commercial wholesaler. 5 luminescent and 3 non-luminescent specimens were maintained in a matured coral reef tank for behavior experiments (670 l capacity including filter-sump and macro-algae filter; 135 cm x 66 cm x 70 cm). Four luminous and 11 non-luminous specimens were kept in a separate tank for structural investigations of light organs. Fish were kept with diurnal reef fishes e.g. surgeonfish, dragonets, hawkfish, damselfish and wrasse. Furthermore a range of invertebrates (including cleaner shrimp *Lysmata amboiensis*) and corals were kept in the tank to simulate the natural environment. *Anomalops katoptron* were kept in a 12 h night and day cycle. Fish were kept in artificial seawater (salt concentration 36 ‰). The water was heated to 26 °C (±1 °C) via a submersible heater. Water quality was maintained via a protein skimmer, aerobic and anaerobic biological denitrification filters, and a macroalgae (*Caulerpa taxifolia* & *Chaetomorpha linum*) filter (AquaMedic, Germany) to control the nitrogen-load induced by the extensive feeding. Every 2 weeks 100 l were replaced with fresh artificial seawater. Fresh seawater was prepared with tap water (purified via reverse-osmosis) and artificial sea salt (Aqua Medic, Germany). Fish were fed with a mix of frozen zooplankton (mysid shrimp and lobster/fish eggs) twice a day *ad libitum*. The frozen mix was defrosted immediately before feeding and administered drop by drop over a time of 20 to 30 minutes. The food was applied into the water current because *A. katoptron* feed only on moving prey. The diet was enriched with fine minced salmon flesh every 3 days. Furthermore the frozen mix was enriched every 3 days with vitamins (Sera, Germany) and salmon oil. Specimens for anatomy experiments were kept in an additional reef tank.

**Identification of individual specimens**

Five luminous and 3 non-luminous (*A. katoptron*) was kept in the coral reef tank 8 months before the behavior experiments started. The fish were observed for at least 30 minutes per day prior to the experiments under the following conditions: (i) feeding under red light illumination (ii) during the night under IR-illumination (iii) during the day in the artificial reef cave. Eight specimens were identified via body size, fin markings, light organ size and non-luminous patches on light organs. Prior to analysis of each experiment the recording was observed in slow motion until a specimen was identified. After identification the recording was rewound to the beginning and the fish position was marked on an overhead-transparency which was taped on the computer screen. The procedure was repeated for each specimen and each recording.

**Anatomy of light organs**

For histological analysis of light organs, specimens of *A. katoptron* (10 cm length) were euthanized with MS-222 (tricaine methanesulfonate) and fixed in 8 % PFA (paraformaldehyde). Light organs were removed and stored at 4°C until processed. Light organs were cryoprotected in 30 % sucrose-solution for at least 24 h and subsequently embedded in Tissue-Tek (Sakura). Fifty µm thin sections were sliced in a sagittal plane. We used PFA auto-fluorescence to outline the anatomical structures of light organs [1]. Photomicrographs were recorded using a Leica TCS SP5 confocal laser scanning microscope (Objectives 10X/0.3 NA & 20X/0.7 NA) and processed with ImageJ (National Institute of Health).

1. Walleser LR, Howard DR, Sandheinrich MB, Gaikowski MP, Amberg JJ. Confocal microscopy as a useful approach to describe gill rakers of Asian species of carp and native filter-feeding fishes of the upper Mississippi River system. J Fish Biol. 2014;85(5): 1777-84.

**S1 Fig**


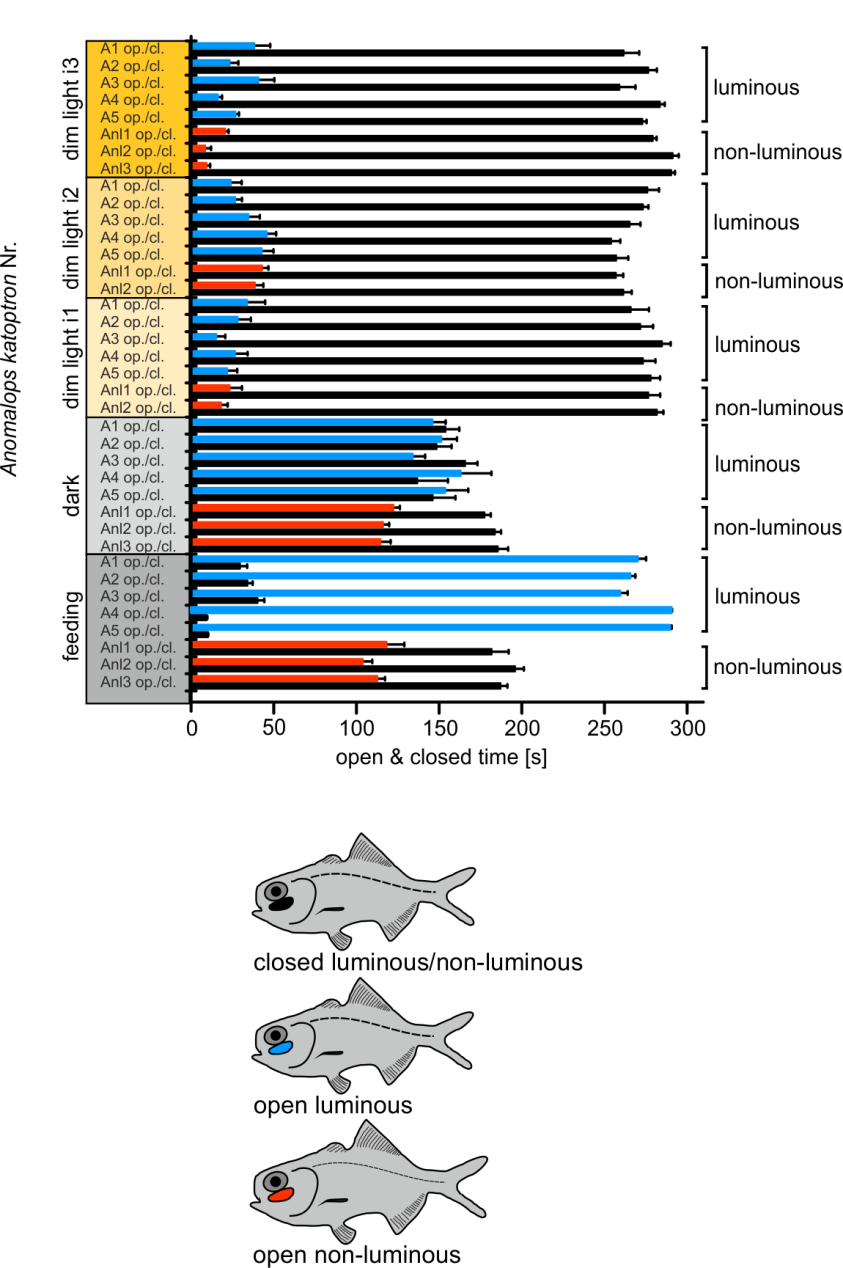


**S1 Fig.** **Blink behaviour in *A. katoptron* during the day (3 dim light conditions), night (darkness) and feeding in darkness (frozen plankton).** (a) Distribution of absolute open/closed time of light organs in *A. katoptron* for specimens with luminous light organs (A1-A5, n=5) and specimens with non-luminous light organs (Anl1-Anl3, n=3) during 3 dim light conditions (3 decreasing light intensity levels i3: 0.63 µW/cm², i2: 0.134 µW/cm² & i1: 0.026 µW/cm², indicated by yellow squares), darkness (indicated by light gray squares) and feeding zooplankton (indicated by dark grey squares). Black bars show closed luminous/non-luminous light organs. Blue bars show luminous open light organs (A1-A5). Red bars show non-luminous open light organs (Anl1-AnL3). Error bars indicate ± SEM. Icons illustrate color coding. Black indicates closed light organs for both luminous and non-luminous light organs. *Blue* indicates luminous open light organs. *Red* indicates non-luminous open light organs.
