# Supplementary figures and images for: The Flashlight Fish Anomalops katoptron Uses Bioluminescent Light to Detect Prey in the Dark
Source: PLoS One. 2017 Feb 8;12(2):e0170489. doi: 10.1371/journal.pone.0170489 (PMC5298212; doi:10.1371/journal.pone.0170489)

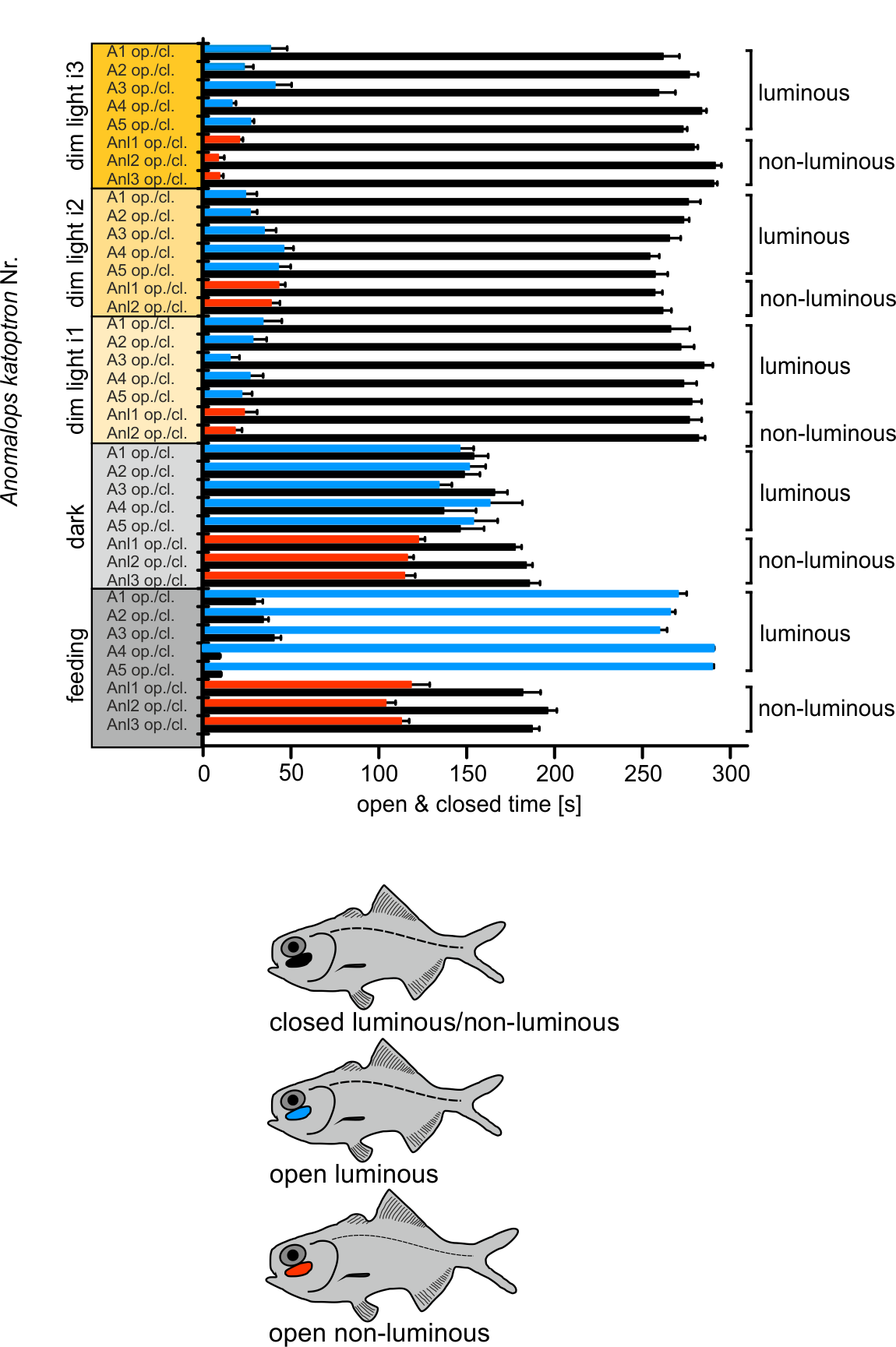

Supplement: S1 Fig — (TIF) [file pone.0170489.s002.tif]
